# Which vertebral levels provide practical HU-based assessment for osteoporosis screening? A comparative analysis from T1 to S2

**DOI:** 10.1186/s13018-026-06952-8

**Published:** 2026-06-23

**Authors:** Ze Zhou, Yan-Xiang Hou, Xin-Yu Dong, Jia-Qi Wang, Lu-Ping Zhou, Yu Chen, Pei-Lin Jin, Hua-Qing Zhang, Chong-Yu Jia, Liang Kang, Hui-Ya Ma, Cai-Liang Shen, Ren-Jie Zhang

**Affiliations:** 1 Department of Orthopedics and Spine Surgery, The First Affiliated Hospital of Anhui Medical University, 210 Jixi Road, Hefei, 230022 Anhui China; 2 Laboratory of Spinal and Spinal Cord Injury Regeneration and Repair, The First Affiliated Hospital of Anhui Medical University, Hefei, 230022 Anhui China; 3 Anhui Province Research Center for the Clinical Application of Digital Medical Technology, The First Affiliated Hospital of Anhui Medical University, Hefei, 230022 Anhui China; 4 Health Management & Checkup Center, The First Affiliated Hospital of Anhui Medical University, 210 Jixi Road, Hefei, 230022 Anhui China; 5 Department of Epidemiology and Biostatistics, School of Public Health, Anhui Medical University, 81 Meishan Road, Hefei, 230032 China

**Keywords:** Thoracic vertebrae, Lumbar vertebrae, Bone mineral density, Hounsfield units, Osteoporosis

## Abstract

**Objective:**

To identify practical vertebral HU measurement strategies for opportunistic bone mineral density (BMD) screening using routine CT.

**Methods:**

Seven anatomical segmental combinations were defined: upper thoracic (T1–T4, UT), mid-thoracic (T5–T8, MT), lower thoracic (T9–T12, LT), thoracolumbar (T11–L2, TL), upper lumbar (L1–L2, UL), lower lumbar (L3–L5, LL), and sacral (S1–S2, SA). Correlations between HU and DEXA-derived T-score were analyzed for both single vertebrae and multi-segment combinations, using the full-segment average (T1–S2, TS) as reference. Stepwise linear regression across 19 vertebrae generated an exploratory combination (EC) model. ROC analysis and age–sex subgroup analyses were performed.

**Results:**

A total of 300 patients were enrolled (150 males, 150 females; mean age 54.94 ± 14.39 years). Both single- and multi-segment measurements correlated positively with T-score. TS showed the strongest correlation (*r* = 0.799) and highest predictive performance (AUC = 0.914). Among single vertebrae, T5 showed the strongest correlation (*r* = 0.785). Among anatomical combinations, TL showed one of the strongest correlations (*r* = 0.787) and favorable predictive performance (AUC = 0.912). Subgroup analyses showed stable TL performance across sex groups, whereas the strongest correlation and predictive performance were observed in the 51–60-year age subgroup.

**Conclusion:**

Both single- and multi-segment HU measurements were effective for BMD assessment. The TL segment represents a practical HU-based assessment approach for opportunistic osteoporosis screening on routine CT, with stable predictive performance and practical clinical value.

**Supplementary Information:**

The online version contains supplementary material available at https://doi.org/10.1186/s13018-026-06952-8.

## Introduction

Osteoporosis is a systemic metabolic bone disease characterized by reduced bone mass, destruction of bone microarchitecture, and increased bone fragility. It has become a major public health challenge in aging societies [1]. Its pathogenesis is complex and often associated with estrogen deficiency, calcium–phosphorus metabolism disorders, vitamin D deficiency, and various chronic diseases [2]. Global statistics indicate that approximately 200 million individuals are affected by osteoporosis. Among people over 50 years old, about one-third of women and one-fifth of men will sustain an osteoporotic fracture (i.e., fragility fracture), and roughly 9 million new cases occur annually [3]. Thoracolumbar fractures are the most common type, representing approximately 50% of all fragility fractures [4]. These fractures not only cause chronic pain and spinal deformity but also markedly increase the risk of subsequent fractures [5], creating a substantial burden on healthcare systems and society.

The pathological changes of osteoporosis primarily occur in trabecular bone and include thinning, disruption, and loss of trabeculae, often accompanied by multiple microfractures. Dual-energy X-ray absorptiometry (DEXA) remains the gold standard for measuring bone mineral density (BMD) and for the clinical assessment of osteoporosis [6]. However, DEXA is a two-dimensional projection technique, and its measurements include vertebral trabecular bone as well as cortical structures such as spinous processes, pedicles, and facet joints. Extra-spinal high-density structures, such as aortic calcification, residual bowel contrast agents, and pancreatic calcifications, may also interfere with the measurements. This can lead to falsely elevated BMD and reduced diagnostic accuracy [7].

Computed tomography (CT) is widely used in routine clinical practice. Opportunistic BMD assessment can be performed on existing CT images by drawing regions of interest (ROI) within vertebral trabecular bone to obtain Hounsfield units (HU) without additional radiation exposure or cost [8–10]. More recently, quantitative CT-derived analysis and artificial intelligence-assisted vertebral attenuation assessment have further expanded the role of routine CT in opportunistic osteoporosis screening by improving measurement standardization and efficiency [11, 12]. Previous studies have demonstrated that vertebral HU can assess BMD and predict complications of degenerative lumbar spine diseases [13, 14]. However, most existing studies have evaluated the lumbar spine [15–17], and systematic comparisons of HU across TL segments remain limited. HU differ across thoracic and lumbar levels, and their predictive performance and cut-offs for osteoporosis also vary. At present, no consensus exists regarding which single- or multi-segment measurement provides the most clinically practical performance for BMD assessment and osteoporosis prediction.

Although previous studies have demonstrated the value of selected lumbar or thoracolumbar vertebrae, particularly L1 or T11–L2, for opportunistic osteoporosis assessment [9, 15], most investigations have focused on isolated vertebral levels or limited lumbar segments rather than performing a systematic comparison across the full thoracic-to-sacral spine. In particular, evidence remains limited regarding the relative performance of single vertebrae, clinically defined anatomical combinations, and statistically selected vertebral combinations within a unified analytical framework.

Therefore, rather than merely reconfirming the value of the thoracolumbar region, the present study provides a whole-spine comparative evaluation across all 19 vertebrae from T1 to S2, integrating anatomical segment combinations, regression-derived selection, and age–sex stratified analyses within a unified analytical framework. Based on this framework, we aimed to identify vertebral HU measurement strategies that balance predictive performance, stability, and clinical practicality for opportunistic bone mineral density screening using routine CT.

## Materials and methods

### Sample selection

This single-center retrospective cohort study consecutively enrolled patients who underwent both thoracic and lumbar CT and DEXA at the First Affiliated Hospital of Anhui Medical University between June 2019 and June 2023. Inclusion criteria were: [1] age between 31 and 80 years; [2] completion of thoracic and lumbar CT (slice thickness ≤ 1.25 mm) and DEXA within one week; and [3] CT images free of metal artifacts. Patients older than 80 years were not included because the available retrospective sample in this age range was limited and did not support balanced subgroup stratification by age and sex. Exclusion criteria were: [1] history of spinal surgery; [2] presence of burst fractures, metastatic tumors, or hematologic disorders; and [3] diagnosis of metabolic bone disease. Exclusion was based on available clinical records and imaging findings at the time of retrospective review. Patients were stratified by age (per decade) and sex, yielding 10 age–sex subgroups. Thirty patients were randomly selected from each subgroup, resulting in a final cohort of 300 patients. This retrospective study was approved by the Institutional Review Board (approval No. PJ2023-12-59). Owing to the retrospective design and the use of anonymized data, written informed consent was waived.

### DEXA measurement of BMD

DEXA was performed using a Lunar Prodigy bone densitometer (GE Medical Systems Lunar Madison, Fairfield, USA) to measure BMD of the lumbar spine (L1–L4) and total hip. Scans were conducted at the Health Examination Center of the First Affiliated Hospital of Anhui Medical University. According to World Health Organization criteria, BMD status was categorized as osteoporosis (T-score ≤ − 2.5), osteopenia (–2.5 < T-score < − 1.0), or normal BMD (T-score ≥ − 1.0). DEXA output included BMD (g/cm²) and corresponding T-scores for routinely assessed skeletal sites. In the present study, osteoporosis classification was based on the average L1–L4 T-score, which was the consistently available reference measure across the full cohort.

### CT measurement of HU

All CT scans were performed using a 64-slice GE CT scanner (GE Discovery CT750 HD) under routine institutional acquisition protocols, using the same scanner platform and reconstruction workflow, with reconstructed slice thickness ≤ 1.25 mm. HU were measured on a post-processing workstation. It should be noted that T1–S2 vertebral images were not obtained from a single whole-spine CT scan. Instead, these images were derived from the routine clinical imaging protocol used in our department, in which thoracic CT (covering T1–T12) and lumbar CT (covering L1–S2) were both available for eligible patients, allowing acquisition of data for all 19 vertebrae.

**Fig. 1:**
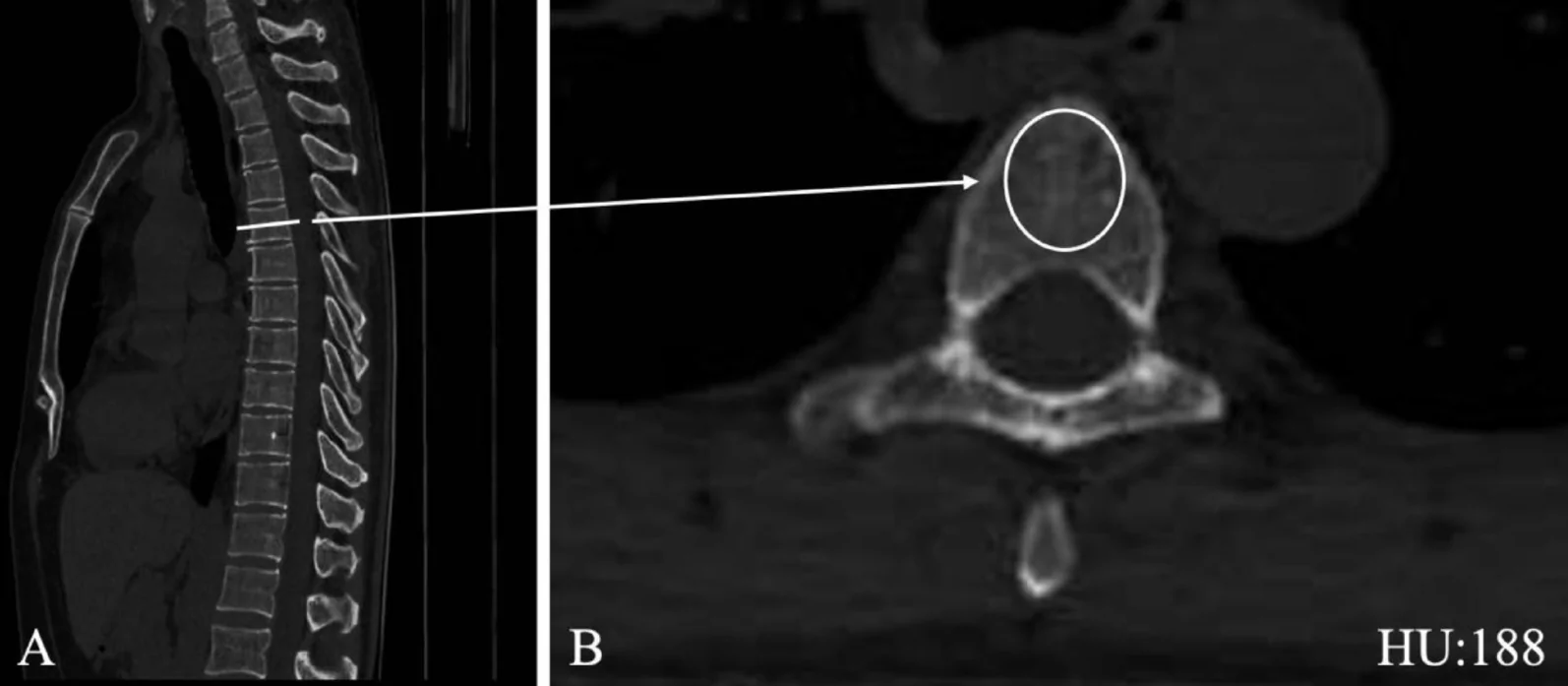
Panel** A**: Mid-sagittal CT reconstruction of the thoracic spine in a 34-year-old male, showing the full thoracic vertebral sequence. Panel** B**: Axial CT slice of the T5 vertebral body. An elliptical ROI, outlined in white, is centered within the trabecular compartment whereas excluding cortical bone and basivertebral venous structures. The same ROI placement method was applied to all vertebrae from T1 to S2 on their respective axial slices

Following established methodologies, HU were measured by placing the largest possible elliptical region of interest (ROI) within the vertebral cancellous bone at the mid-vertebral level, parallel to the endplate. Because vertebral size varies across vertebral levels and individuals, a fixed ROI area was not imposed; instead, a standardized placement principle was used to maximize trabecular sampling while preserving exclusion of cortical margins and focal abnormalities. Cortical bone, basivertebral veins, and focal lesions were carefully excluded (Fig. 1). If a vertebral body exhibited fragility fractures or severe degenerative changes, the average HU of the two adjacent vertebrae was used as a substitute.

Two spinal specialists independently outlined the ROI under blinded conditions. Inter-rater reliability was excellent (intraclass correlation coefficient, ICC = 0.92; 95% CI 0.88–0.95). When the difference between the two measurements exceeded 15 HU, a third physician was consulted. A discrepancy threshold of 15 HU was used as a practical criterion to identify potentially meaningful interobserver disagreement and trigger third-reader adjudication when measurement differences were considered likely to affect vertebral attenuation assessment. To assess intra-rater reproducibility, one physician repeated the measurements in 30 randomly selected cases four weeks later. The intra-rater ICC was 0.94 (95% CI 0.91–0.97), indicating high repeatability and stability of HU measurements.

### Anatomical segmentation and stepwise regression model

To systematically identify clinically representative vertebral groupings, the T1–S2 region was divided into seven clinically commonly used anatomical segment groups: upper thoracic (T1–T4, UT), mid-thoracic (T5–T8, MT), lower thoracic (T9–T12, LT), thoracolumbar (T11–L2, TL), upper lumbar (L1–L2, UL), lower lumbar (L3–L5, LL), and sacral (S1–S2, SA). Stepwise linear regression was then performed using HU of all 19 vertebrae (T1–S2) as independent variables and the DEXA-derived T-score as the dependent variable to construct an exploratory combination (EC) model. This exploratory model was subsequently compared with the anatomical segment groups as a non-anatomical reference for BMD assessment performance.

### Statistical analysis

All statistical analyses were conducted using SPSS version 25.0 (SPSS Inc., Chicago, IL, USA). Descriptive statistics for continuous variables (age, BMI, hypertension, diabetes, smoking, and alcohol consumption) are presented as mean ± standard deviation (SD). Categorical variables were compared using the Chi-square test. Using the average HU of the full T1–S2 segment (TS) as the reference, Spearman correlation coefficients were calculated to assess correlations between HU (single or multi-segment) and the DEXA-derived T-score. Correlation strength was classified as high (*r* = 0.50–1.00), moderate (*r* = 0.30–0.49), or low (*r* < 0.30). To evaluate predictive performance for osteoporosis, receiver operating characteristic (ROC) curve analysis was performed for both single and multi-segment HU combinations. The DeLong test was used to compare differences in area under the curve (AUC) values between segments. The optimal cut-off was identified using the Youden index. Subgroup analyses were conducted for the TL segment by age (per decade) and sex, and correlation coefficients (r), AUC values, and optimal cut-off values were reported for each subgroup where applicable.

To account for multiple comparisons, a total of 27 independent correlation tests (19 single segments + 7 multi-segment combinations + 1 full-segment combination) were performed. The Bonferroni correction was applied, setting the adjusted significance level at α = 0.05 / 27 ≈ 0.002. A *p* < 0.002 was therefore considered statistically significant.

## Results

### Patient demographics and baseline characteristics

A total of 300 patients were included in this study (150 males and 150 females). The mean age was 54.94 ± 14.39 years, with males at 54.77 ± 14.64 years and females at 55.11 ± 14.17 years. There was no statistically significant age difference between sexes (*p* = 0.857). DEXA measurements showed that BMD was 1.00 ± 0.24 g/cm², overall, with males at 1.06 ± 0.23 g/cm² and females at 0.95 ± 0.24 g/cm², demonstrating a statistically significant difference (*p* < 0.05). The mean T-score was − 0.76 ± 1.99, with males at − 0.18 ± 1.89 and females at − 1.35 ± 1.92, also demonstrating a statistically significant difference (*p* < 0.05). The mean HU of T1–S2 was 133.27 ± 55.51, with males at 136.06 ± 47.77 and females at 130.47 ± 62.34, with no statistically significant difference (*p* = 0.060) (Table 1). Multivariate logistic regression showed that hypertension, diabetes, smoking, and alcohol consumption did not significantly affect bone density status (*p* > 0.05).

**Table 1:** Comparison of general characteristics and BMD data (*n* = 300)

Variables	Total (*n* = 300)	Male (*n* = 150)	Female (*n* = 150)	*P* value
Age	54.94 ± 14.39	54.77 ± 14.64	55.11 ± 14.17	0.857
BMI (kg/m^2^)	23.53 ± 3.36	23.55 ± 2.88	23.51 ± 3.79	0.435
Hypertension	52 (17.3%)	36 (24.0%)	16 (10.7%)	0.002
Diabetes	16 (5.3%)	8 (5.3%)	8 (5.3%)	1.000
Smoking	43 (14.3%)	42 (28%)	1 (0.7%)	< 0.001
Alcohol consumption	34 (11.3%)	33 (22%)	1 (0.7%)	< 0.001
HU (T1–S2)	133.27 ± 55.51	136.06 ± 47.77	130.47 ± 62.34	0.060
BMD (g/cm^2^)	1.00 ± 0.24	1.06 ± 0.23	0.95 ± 0.24	< 0.001
T-score	− 0.76 ± 1.99	− 0.18 ± 1.89	− 1.35 ± 1.92	< 0.001

### Distribution characteristics of TL HU

**Fig. 2:**
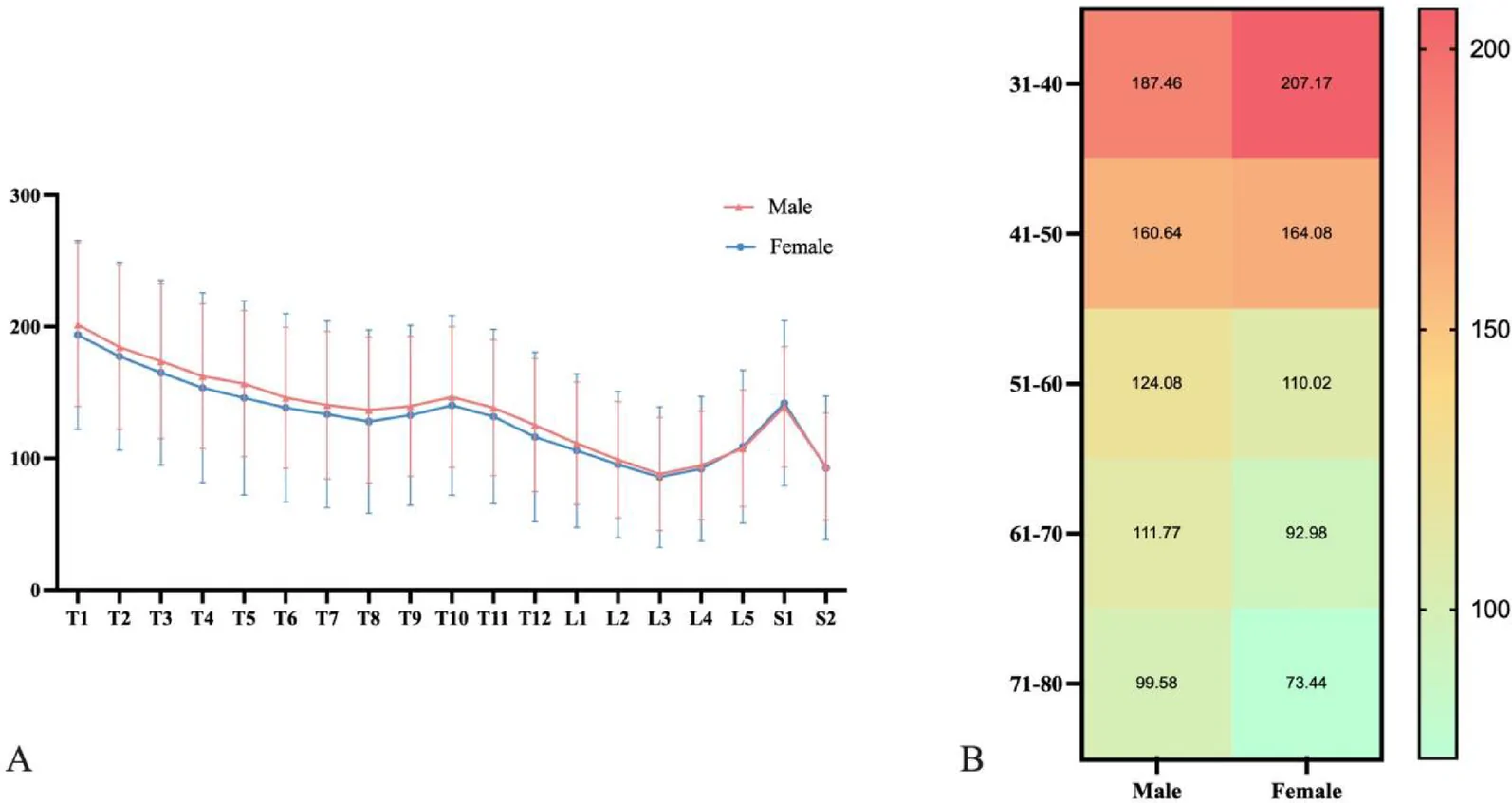
Panel** A**: Line graph showing mean HU for each vertebra (T1–S2) stratified by sex. Panel** B**: Comparison of HU gradients between males and females stratified by age

As shown in Fig. 2A, HU from T1 to S2 exhibited a “decline–rebound” pattern: peaking at T1 (197.69 ± 67.13), descending to a local high at T10 (143.39 ± 61.37), continuing downward to the lowest point at L3 (86.95 ± 48.27), and rising again toward S1 (140.45 ± 54.74) (Supplementary Table 1). Gender-stratified curves are presented in Fig. 2B.

Linear regression revealed age-related annual decreases of 2.23 HU (95% CI − 2.61 to − 1.84, *p* < 0.001) in males and 3.47 HU (95% CI − 3.91 to − 3.03, *p* < 0.001) in females. Two-way ANOVA (age × sex) showed a significant main effect of age (F = 10.347, *p* < 0.001), no main effect of sex (F = 0.354, *p* = 0.553), and a significant age–sex interaction (F = 2.401, df = 38, *p* < 0.001), indicating that the rate of age-related HU decline differs between males and females.

### Correlation between single- and multi-segment TL HU and T-score

The full-segment (TS) average showed the strongest correlation with T-score (*r* = 0.799). Among single segments, all vertebrae from T1 to S2 showed strong associations (*r* ≥ 0.50), with the highest correlation at T5 (*r* = 0.785) and, within the lumbar spine, at L1 (*r* = 0.776) (Supplementary Table 2).

Given the high collinearity among adjacent vertebral HU values, stepwise linear regression was used for exploratory variable selection. T5, L1, and T6 were retained, yielding the EC model (adjusted R^2^ = 0.594). In this model, the standardized β for T6 was − 0.819, indicating a suppressor effect that alleviated multicollinearity introduced by T5, whereas T5 and L1 carried the primary trabecular-density information (β = 1.224 and 0.368, respectively).

**Fig. 3:**
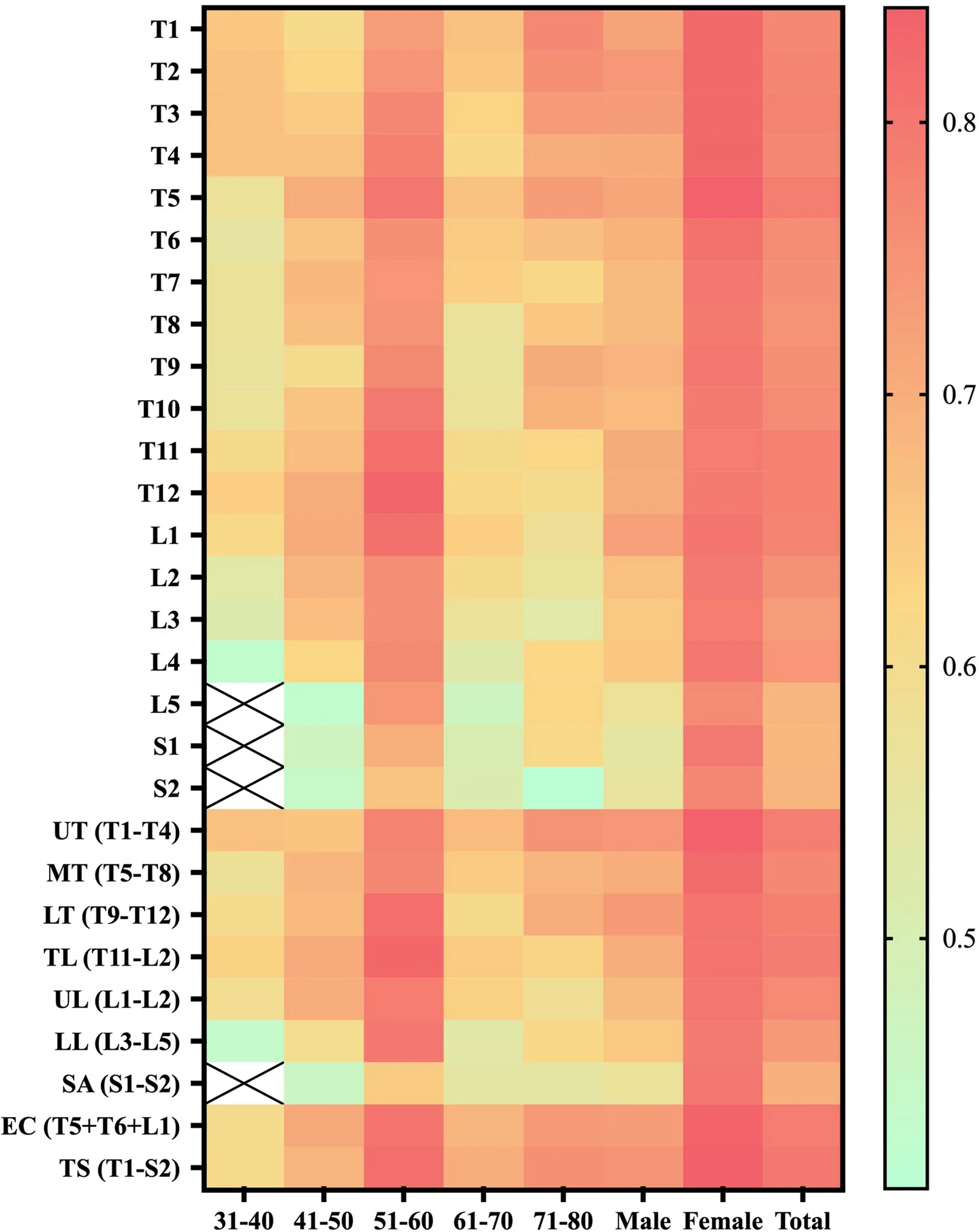
Age- and sex-stratified heat map of correlation coefficients between HU of single (T1–S2) and multi-segment combinations and T-score

Among multi-segment anatomical groups, all correlations remained strong (*r* ≥ 0.50). The EC model also showed high correlation with T-score (*r* = 0.792), followed closely by the TL segment (T11–L2, *r* = 0.787), while the SA segment showed the lowest correlation (*r* = 0.694) (Fig. 3).

### Diagnostic performance of TL HU for osteoporosis

**Fig. 4:**
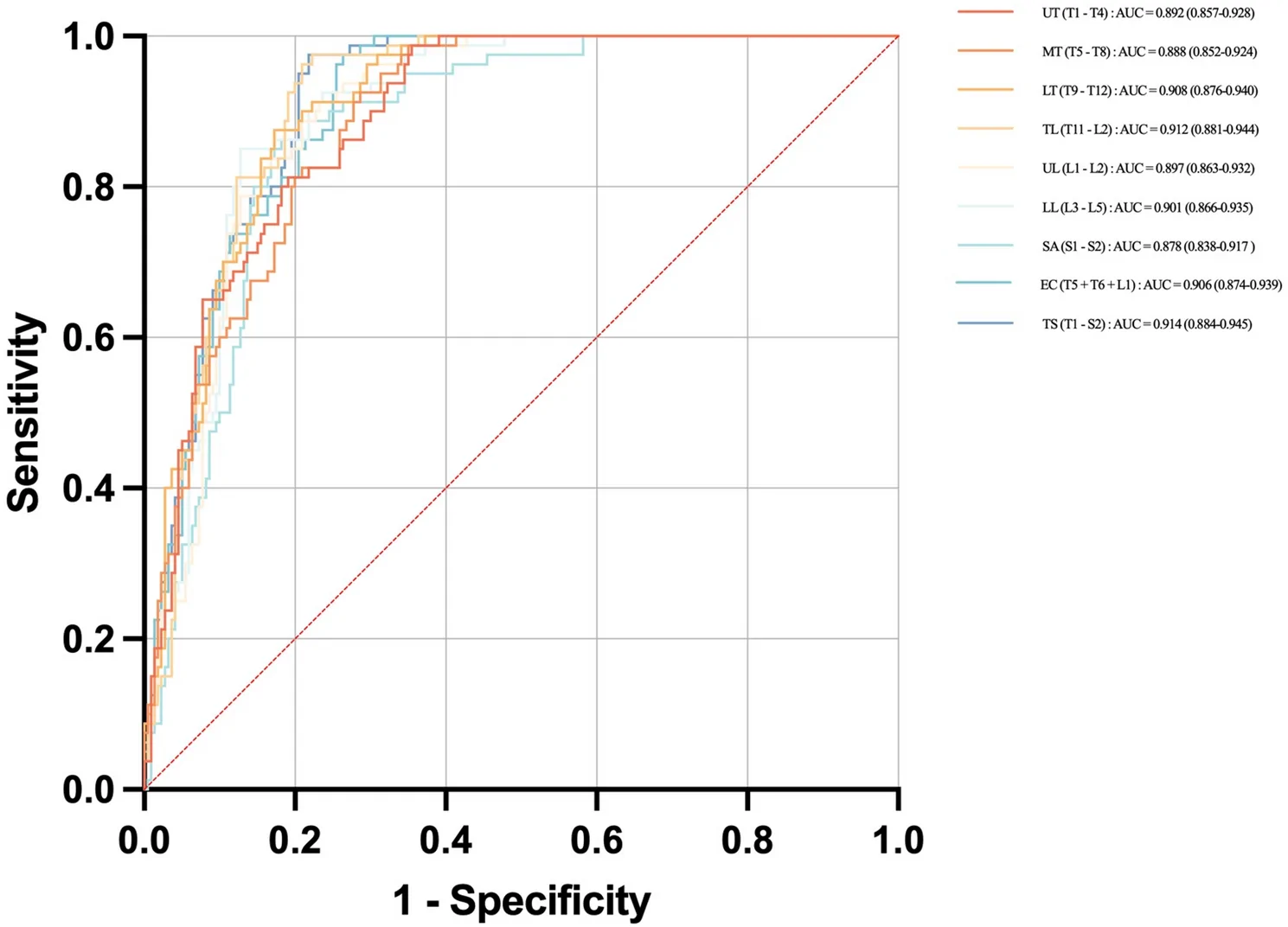
Performance metrics of multi-segment HU combinations in predicting osteoporosis

The full-segment (T1–S2) HU achieved the highest AUC of 0.914 for osteoporosis prediction, with an optimal cut-off of 110.3 HU (sensitivity 97.50%, specificity 78.18%). Among single vertebrae, AUC values ranged from 0.862 to 0.912, with T11 showing one of the highest AUC values (0.912; cut-off 100.5 HU; sensitivity 88.75%; specificity 83.64%). Among multi-segment combinations, the TL segment (T11–L2) showed similarly high predictive performance (AUC = 0.912), with stable sensitivity and specificity (Fig. 4). The EC model (T5 + T6 + L1) achieved an AUC of 0.906, only 0.007 lower than the full-segment model (Table 2). DeLong tests revealed no statistically significant differences between the anatomical segment combinations (all *p* > 0.05).

**Table 2:** Performance indicators of multi-segment HU combinations for osteoporosis prediction

Segment	AUC (95% CI)	cut-off (HU)	Sensitivity (%)	Specificity (%)	*p*-value
UT (T1–T4)	0.892 (0.857–0.928)	166.6	98.75	64.55	< 0.001
MT (T5–T8)	0.888 (0.852–0.924)	121.9	92.50	71.36	< 0.001
LT (T9–T12)	0.908 (0.876–0.940)	100.6	87.50	82.73	< 0.001
TL (T11–L2)	0.912 (0.881–0.944)	93.1	96.25	79.09	< 0.001
UL (L1–L2)	0.897 (0.863–0.932)	82.3	91.25	76.82	< 0.001
LL (L3–L5)	0.901 (0.866–0.935)	64.2	85.00	87.27	< 0.001
SA (S1–S2)	0.878 (0.838–0.917)	89.2	86.25	81.82	< 0.001
EC (T5 + T6 + L1)	0.906 (0.874–0.939)	85.8	97.50	73.64	< 0.001
TS (T1–S2)	0.914 (0.884–0.945)	110.3	97.50	78.18	< 0.001

To examine the internal consistency of the TL cut-off, the 93.1 HU cut-off was applied to the full cohort of 300 patients. The resulting confusion matrix was highly consistent with the ROC-derived estimates (Table 3), indicating good internal consistency of this cut-off within the current dataset.

**Table 3:** Observed confusion matrix for osteoporosis prediction using the TL combination at the 93.1 HU threshold (*n* = 300)

Actual Condition	HU ≤ 93.1 (Positive)	HU > 93.1 (Negative)	Total
Osteoporosis (T-score ≤ −2.5)	77 (TP)	3 (FN)	80
Non-osteoporosis (T-score > −2.5)	46 (FP)	174 (TN)	220
Total	123	177	300

TP = True Positive, FP = False Positive, TN = True Negative, FN = False Negative

### Subgroup analysis of TL segment performance

To improve clinical interpretability, subgroup analyses were performed for the TL segment according to sex and age (Table 4). In sex-stratified analysis, the TL segment showed consistently strong correlations with T-score in both males and females, with slightly stronger correlation observed in females (*r* = 0.808 vs. 0.740). Predictive performance remained stable across sexes, with similar AUC (0.899–0.909) and nearly identical optimal cut-off around 93 HU.

**Table 4:** Subgroup analysis of the TL segment (T11–L2) for osteoporosis screening

Segment	Correlation with T-score, *r*	AUC (95% CI)	Optimal cut-off (HU)	Sensitivity (%)	Specificity (%)
Male	0.740	0.909 (0.860–0.959)	93.13	95.65	79.53
Female	0.808	0.899 (0.847–0.950)	93.00	96.49	78.49
31–40	0.633	Not estimable	–	–	–
41–50	0.706	Not estimable	–	–	–
51–60	0.825	0.912 (0.839–0.986)	88.75	95.24	82.05
61–70	0.645	0.808 (0.699–0.917)	93.13	95.24	66.67
71–80	0.630	0.779 (0.655–0.903)	65.37	87.50	64.29

Across age groups, the strongest correlation and highest AUC were observed in the 51–60-year subgroup (*r* = 0.825; AUC = 0.912), whereas predictive performance gradually declined in older groups. ROC analysis was not estimable in the 31–40- and 41–50-year subgroups because no osteoporosis cases were identified in these strata.

## Discussion

With ageing, bone resorption progressively exceeds bone formation, leading to a marked increase in osteoporosis and its complications. Most patients remain undiagnosed until a fragility fracture occurs, suggesting that the actual prevalence is substantially underestimated. The fracture risk among individuals with osteoporosis is approximately 4–6 times higher than among peers without osteoporosis [5], creating substantial socioeconomic burden. Although DEXA is the gold standard for BMD assessment [6], its two-dimensional projection cannot separate cortical from trabecular bone and is easily confounded by degenerative spinal changes and extra-spinal calcification, often producing falsely elevated values. Routine CT therefore provides an important opportunity for opportunistic BMD evaluation; it is sensitive, requires no additional scanning, and allows osteoporosis screening without extra radiation or additional cost [18]. However, most prior investigations focused solely on the lumbar spine, and systematic comparisons across the thoracic-to-sacral region from T1 to S2 using age–sex stratification have been limited. In recent years, opportunistic CT-based bone assessment has expanded beyond simple vertebral HU measurement toward more standardized quantitative CT-derived workflows, including volumetric attenuation assessment, automated vertebral segmentation, and artificial intelligence–assisted opportunistic screening pipelines [11, 12]. These developments have improved measurement reproducibility and further strengthened the role of routine CT in opportunistic osteoporosis screening under non-dedicated imaging conditions. Nevertheless, in routine clinical practice, rapid manual assessment remains common, particularly in settings where automated post-processing is not yet fully integrated. In this context, identification of a limited but stable vertebral region such as T11–L2 remains clinically relevant, because it provides a practical target for both manual opportunistic assessment and future automated implementation.

In this study, HU from T1 to S2 demonstrated a bimodal pattern: descending from T1 to T8, rebounding at T10, continuing downward to the lowest point at L3, and rising again from L4 to S1. This trend is consistent with the TL pattern described by Simion et al. [19]. Xue et al. also reported that thoracic trabecular HU are significantly higher than lumbar HU [9], which aligns with our observations. This distribution corresponds to spinal mechanical loading. The thoracic spine, stabilized by the rib cage, has limited mobility, bears only about 10% of body weight [20], and exhibits minimal degeneration. In contrast, the lumbar spine functions as the key load-bearing transition zone, experiences higher mechanical stress, and shows earlier micro-damage, end-plate sclerosis, and fat infiltration, which explains the lowest HU at L3–L4. By contrast, the sacrum is supported by the pelvic ring. Dynamic X-ray studies have shown that shear strain at L5–S1 is generally lower than at L4–L5 [21], reflecting a relatively low-strain environment that may delay trabecular fatigue damage and mineral loss, thereby accounting for higher HU at S1.

Age is a well-established risk factor for bone loss [22]. After age stratification, T1–S2 HU declined linearly at an overall rate of − 2.83 HU/year. After sex stratification, the decline rate was − 3.47 HU/year in women, approximately 1.6 times that in men (− 2.23 HU/year), consistent with the physiological fall in estrogen after menopause [23]. More importantly, the negative correlation between HU and age (*r* = − 0.732) was stronger than that between T-score and age (*r* = − 0.456), indicating that HU may be more sensitive to age-related bone loss. Two mechanisms may explain this pattern: first, as red marrow converts to yellow marrow, trabecular bone is replaced by fat, causing HU to decrease earlier; second, DEXA measurements include a larger proportion of cortical bone and high-density structures, which can mask early trabecular loss. In addition, the 50–60-year age group exhibited the strongest correlation between HU and T-score, likely because accelerated bone loss, estrogen reduction, and progressive trabecular deterioration during this transitional stage produce clearer discrimination between osteoporosis and non-osteoporosis before degenerative sclerosis becomes more prominent in older groups. In younger groups, the lower prevalence of osteoporosis may reduce discriminatory separation, whereas in older groups increasing structural degeneration may partly weaken alignment between HU and DEXA-derived bone status. This may also explain why predictive performance did not continue to improve in the 61–70-year subgroup despite a higher expected prevalence of osteoporosis, because degenerative sclerosis increasingly affects projection-based DEXA measurements in older individuals. Sex-stratified analysis showed slightly stronger HU–T-score correlation in females than in males, whereas AUC values remained comparable between sexes. This may reflect the more pronounced and hormonally driven trabecular bone loss in women, which can produce clearer linear correspondence between HU and DEXA-derived bone status, while the overall diagnostic stability of the TL segment remained similar in both sexes.

In the single-segment analysis, HU of all T1–S2 vertebrae were strongly correlated with T-score (*r* ≥ 0.50), with T5 showing the highest correlation (*r* = 0.785) and L1 demonstrating the strongest correlation within the lumbar spine (*r* = 0.776). These values are consistent with those reported by Pickhardt et al. (*r* = 0.70) [15], Lee et al. (*r* = 0.72) [24], and Zhang et al. (*r* = 0.74) [16]. T5 and L1 are located in physiologically stable and mechanically representative regions: the mid-thoracic segment and the TL junction. These regions have clear anatomical boundaries and minimal degenerative interference. Their combined use in the EC model provided an exploratory non-anatomical combination with performance close to that of predefined anatomical segments. However, because this regression-derived combination was obtained within the same cohort, it should primarily be interpreted as a data-driven reference rather than a directly transferable measurement strategy. Among multi-segment anatomical groups, the TL segment (T11–L2) also showed high correlation with T-score. The characteristics of this region provide a clear anatomical basis for its performance: T11–T12 are floating-rib vertebrae with greater vertebral height and trabecular area than the mid-thoracic vertebrae, whereas L1–L2 have a thinner cortex, high trabecular content, and relatively delayed marrow fat infiltration. Moreover, these four vertebrae are contiguous and are often captured in a single CT acquisition, reducing variability due to scanning parameters. By contrast, correlations decrease markedly below L4, likely due to increased degenerative change (end-plate sclerosis, osteophytes, vascular calcification) and saturated marrow fat conversion, causing HU to diverge from DEXA-derived bone status.

Our findings showed that all T1–S2 segments had good predictive performance for osteoporosis (AUC 0.862–0.912), with no statistically significant differences between segments based on the DeLong test (*p* > 0.05). This suggests that multiple vertebral levels can provide useful BMD-related information, offering flexibility for clinical measurement. Among single segments, T11 showed one of the highest AUC values (AUC = 0.912), followed by T12 (AUC = 0.911) and L1 (AUC = 0.900), all with nearly identical predictive performance. Notably, T11, T12, and L1 are included within the TL segment (T11–L2), which showed stable and favorable predictive performance among anatomical multi-segment groups (AUC = 0.912) and exhibited a narrower confidence interval, suggesting relatively stable predictive performance. This may reflect three factors: (i) the contiguous anatomy of the TL junction, which facilitates rapid localization and standardized measurement; (ii) the biomechanical importance of T11–L2 as the transition where thoracic stability meets lumbar load-bearing; and (iii) the frequent visualization of these segments on routine thoraco-abdominal CT, supporting wide clinical applicability. Recent opportunistic CT studies have likewise reported that thoracolumbar vertebrae provide stable predictive performance because they combine relatively preserved trabecular structure with frequent inclusion on routine thoraco-abdominal CT [25]. Compared with these studies, the present analysis further suggests that this stability remains preserved when TL is evaluated against all thoracic-to-sacral vertebral levels within the same cohort and under unified measurement conditions. However, unlike most previous studies focusing on isolated lumbar levels or limited thoracolumbar segments, the present study extends comparison across all 19 vertebrae from T1 to S2 and integrates anatomical combinations, regression-derived selection, and subgroup analysis within a unified framework.

When using HU for BMD assessment, averaging more vertebrae helps reduce measurement error. As demonstrated in this study, the full-segment (TS) approach effectively minimizes fluctuations caused by local factors such as degeneration and measurement error, achieving the strongest correlation with DEXA T-score and consistently high predictive performance. However, whole-spine CT is not always available, and measuring all vertebrae can be time-consuming. The TL segment showed predictive performance comparable to the full-segment approach, while the EC model should be interpreted as an exploratory non-anatomical reference. The EC model uses only three vertebrae and may serve as an exploratory non-anatomical measurement alternative, although further individualized optimization would require larger clinically stratified datasets. In routine clinical practice, the TL segment remains attractive because it spans a continuous and easily identifiable region that is frequently visible on thoracic, abdominal, and preoperative spinal CT examinations, allowing opportunistic osteoporosis assessment without dedicated additional imaging. Recent advances in automated vertebral segmentation and artificial intelligence-assisted HU extraction may further improve efficiency and reproducibility when multiple vertebral levels are assessed simultaneously, particularly when a predefined clinically stable region such as T11–L2 is selected as the measurement target [26, 27]. Future multicenter, large-scale studies are warranted to further validate and expand the use of this approach.

This study has several limitations. First, as a single-center retrospective study based on patients undergoing thoracic and lumbar CT together with DEXA in a spine-specialized clinical setting, selection bias cannot be completely excluded despite predefined exclusion criteria, which may limit generalisability. Second, lumbar spine T-score was used as the reference standard because it was consistently available across the full cohort; however, as a projection-based method, DEXA may overestimate bone mineral density in the presence of degenerative sclerosis, osteophytes, endplate changes, and vascular calcification. Therefore, the reported correlations and predictive performance of vertebral HU may partly reflect agreement with a clinically practical but imperfect reference standard, and hip T-score may provide an important complementary reference in future validation studies. Third, the regression-derived EC model should be interpreted as exploratory, because no internal resampling or external validation was performed and some degree of model instability cannot be excluded. Finally, HU were derived from routine clinical CT without phantom-based calibration, and although all scans were acquired on the same scanner platform, residual variation related to reconstruction parameters remains possible. In addition, the proposed cut-off values were evaluated within the same cohort and still require independent multicenter external validation before broader clinical adoption.

## Conclusion

This study demonstrates that HU measured from routine CT can provide effective support for opportunistic BMD assessment. Among the evaluated anatomical regions, the TL segment (T11–L2) showed stable predictive performance and clinical practicality for HU-based assessment, although no statistically significant superiority over adjacent segments was observed. It also maintained stable performance across age and sex strata and may therefore serve as a practical vertebral region for opportunistic osteoporosis assessment in routine clinical imaging.

## Supplementary Information

Below is the link to the electronic supplementary material.

Supplementary Material 1

Supplementary Material 2

Supplementary Material 3

## Data Availability

The data and materials used in this study are available from the corresponding authors (Cai-Liang Shen and Ren-Jie Zhang) upon reasonable request.
