# Supplementary material for: Which vertebral levels provide practical HU-based assessment for osteoporosis screening? A comparative analysis from T1 to S2
Source: J Orthop Surg Res. 2026 Jun 23;21:565. doi: 10.1186/s13018-026-06952-8 (PMC13621712; doi:10.1186/s13018-026-06952-8)
Supplement: Supplementary file 1 [file 13018_2026_6952_MOESM1_ESM.docx]

Supplementary Table 1. Age- and sex-stratified distribution of single- and multi-segment HU (mean ± SD, HU)

| Segment | Age-Group | | | | | Sex-Group | | Total |
| --- | --- | --- | --- | --- | --- | --- | --- | --- |
|  | 31-40 | 41-50 | 51-60 | 61-70 | 71-80 | Male | Female |  |
| T1 | 267.45±60.64 | 230.05±63.62 | 180.15±48.51 | 159.52±32.85 | 151.27±41.01 | 201.61±62.20 | 193.77±71.72 | 197.69±67.13 |
| T2 | 249.57±61.45 | 212.18±64.70 | 164.68±51.72 | 143.42±31.13 | 134.93±38.79 | 184.42±62.29 | 177.49±71.43 | 180.96±67.00 |
| T3 | 238.43±57.96 | 200.25±59.07 | 151.53±49.49 | 133.38±31.67 | 123.60±36.75 | 173.79±58.81 | 165.09±70.10 | 169.44±64.74 |
| T4 | 226.80±57.40 | 188.58±57.39 | 140.04±46.79 | 123.26±33.35 | 111.34±37.50 | 162.40±54.98 | 153.61±72.02 | 158.00±64.11 |
| T5 | 224.92±52.42 | 183.96±54.49 | 132.53±47.87 | 115.86±35.76 | 99.42±37.62 | 156.75±55.52 | 145.93±73.75 | 151.34±65.39 |
| T6 | 214.65±51.93 | 173.90±52.54 | 122.65±44.80 | 107.19±35.48 | 92.86±33.57 | 146.07±53.67 | 138.43±71.66 | 142.25±63.32 |
| T7 | 209.22±55.76 | 170.32±56.20 | 116.03±44.19 | 102.41±32.40 | 86.80±31.62 | 140.44±55.97 | 133.48±71.02 | 136.96±63.93 |
| T8 | 204.39±49.68 | 163.51±57.41 | 111.94±43.92 | 97.22±32.14 | 84.43±32.62 | 136.75±55.47 | 127.84±69.50 | 132.30±62.93 |
| T9 | 207.24±49.40 | 167.23±54.19 | 116.87±42.14 | 102.35±30.65 | 87.09±28.91 | 132.75±68.43 | 133.01±68.27 | 136.15±61.29 |
| T10 | 213.80±50.64 | 176.84±51.77 | 124.73±43.04 | 110.05±28.94 | 91.53±27.46 | 146.52±53.60 | 140.25±68.30 | 143.39±61.37 |
| T11 | 201.01±52.03 | 168.06±46.40 | 119.41±43.25 | 104.48±28.72 | 82.29±27.79 | 138.39±51.79 | 131.71±66.18 | 135.05±59.42 |
| T12 | 172.59±48.70 | 152.85±44.76 | 105.77±42.45 | 92.00±29.49 | 68.00±28.32 | 125.30±50.47 | 116.21±64.42 | 120.76±57.95 |
| L1 | 166.45±43.07 | 137.35±45.16 | 94.95±38.09 | 82.97±27.39 | 61.30±25.57 | 111.33±46.54 | 105.87±58.42 | 108.60±52.80 |
| L2 | 152.25±38.40 | 122.92±42.43 | 86.13±38.48 | 72.83±26.98 | 51.40±25.75 | 98.89±44.23 | 95.33±55.60 | 97.11±50.18 |
| L3 | 139.85±37.63 | 111.00±41.94 | 67.67±36.37 | 63.47±27.00 | 43.75±24.12 | 88.13±42.83 | 85.77±53.28 | 86.95±48.27 |
| L4 | 146.38±39.02 | 114.92±38.34 | 82.85±39.91 | 69.18±28.60 | 53.20±28.49 | 94.60±41.16 | 91.01±54.88 | 93.31±48.44 |
| L5 | 161.85±44.54 | 130.42±42.97 | 97.58±44.57 | 81.92±32.30 | 69.18±28.43 | 107.50±44.35 | 108.88±57.99 | 108.19±51.54 |
| S1 | 193.42±39.82 | 164.02±45.87 | 132.23±47.84 | 114.83±36.25 | 97.75±31.45 | 139.00±45.69 | 141.90±62.62 | 140.45±54.74 |
| S2 | 146.12±42.53 | 116.47±36.48 | 81.05±35.56 | 68.77±27.23 | 53.58±28.60 | 93.71±40.78 | 92.68±54.54 | 93.20±48.08 |
| UT (T1–T4) | 246.90±56.89 | 208.87±59.45 | 160.09±49.47 | 140.67±32.11 | 130.28±37.90 | 181.48±58.94 | 173.25±70.86 | 177.36±65.19 |
| MT (T5–T8) | 213.29±51.41 | 172.92±54.55 | 120.79±44.55 | 105.67±33.12 | 90.87±33.06 | 145.00±54.64 | 136.42±71.06 | 140.71±63.42 |
| LT (T9–T12) | 201.76±49.05 | 166.31±47.70 | 116.74±41.52 | 102.42±28.11 | 82.25±26.83 | 137.50±51.18 | 130.30±66.02 | 133.90±59.08 |
| TL (T11–L2) | 176.22±44.04 | 145.29±43.75 | 101.56±39.22 | 88.07±27.03 | 65.75±25.37 | 118.48±47.33 | 112.28±60.33 | 115.38±54.22 |
| UL (L1–L2) | 159.35±40.12 | 130.33±43.83 | 90.54±37.91 | 77.90±26.74 | 56.35±38.97 | 105.11±45.06 | 100.60±56.76 | 102.86±51.21 |
| LL (L3–L5) | 148.65±38.97 | 118.55±39.20 | 85.70±39.00 | 71.52±27.48 | 55.38±25.75 | 96.37±40.95 | 95.55±54.46 | 95.96±48.10 |
| SA (S1–S2) | 169.14±46.59 | 140.45±42.52 | 106.70±41.13 | 91.16±29.41 | 75.67±27.77 | 115.23±42.57 | 118.01±58.25 | 116.62±50.95 |
