# Supplementary material for: Which vertebral levels provide practical HU-based assessment for osteoporosis screening? A comparative analysis from T1 to S2
Source: J Orthop Surg Res. 2026 Jun 23;21:565. doi: 10.1186/s13018-026-06952-8 (PMC13621712; doi:10.1186/s13018-026-06952-8)
Supplement: Supplementary file 2 [file 13018_2026_6952_MOESM2_ESM.docx]

Supplementary Table 2. Correlation coefficients between single- and multi-segment HU and T-score

|  | Age-Group | | | | | Sex-Group | | Total |
| --- | --- | --- | --- | --- | --- | --- | --- | --- |
| Segment | 31-40 | 41-50 | 51-60 | 61-70 | 71-80 | Male | Female |  |
| T1 | 0.655 | 0.608 | 0.732 | 0.664 | 0.770 | 0.720 | 0.824 | 0.769 |
| T2 | 0.662 | 0.627 | 0.747 | 0.653 | 0.755 | 0.741 | 0.824 | 0.775 |
| T3 | 0.667 | 0.644 | 0.772 | 0.631 | 0.737 | 0.730 | 0.824 | 0.777 |
| T4 | 0.663 | 0.664 | 0.784 | 0.616 | 0.701 | 0.705 | 0.826 | 0.772 |
| T5 | 0.574 | 0.700 | 0.803 | 0.662 | 0.731 | 0.714 | 0.839 | 0.785 |
| T6 | 0.551 | 0.660 | 0.754 | 0.646 | 0.669 | 0.690 | 0.810 | 0.761 |
| T7 | 0.569 | 0.682 | 0.744 | 0.640 | 0.617 | 0.675 | 0.799 | 0.756 |
| T8 | 0.567 | 0.669 | 0.750 | 0.564 | 0.655 | 0.675 | 0.794 | 0.750 |
| T9 | 0.560 | 0.604 | 0.766 | 0.563 | 0.704 | 0.692 | 0.798 | 0.755 |
| T10 | 0.569 | 0.660 | 0.795 | 0.571 | 0.690 | 0.674 | 0.793 | 0.764 |
| T11 | 0.609 | 0.672 | 0.814 | 0.610 | 0.621 | 0.703 | 0.791 | 0.782 |
| T12 | 0.641 | 0.701 | 0.833 | 0.618 | 0.601 | 0.700 | 0.793 | 0.783 |
| L1 | 0.612 | 0.706 | 0.811 | 0.641 | 0.583 | 0.727 | 0.805 | 0.776 |
| L2 | 0.532 | 0.686 | 0.758 | 0.609 | 0.566 | 0.666 | 0.795 | 0.753 |
| L3 | 0.516 | 0.670 | 0.756 | 0.572 | 0.532 | 0.651 | 0.789 | 0.733 |
| L4 | 0.422 | 0.621 | 0.765 | 0.524 | 0.619 | 0.653 | 0.799 | 0.743 |
| L5 | - | 0.422 | 0.742 | 0.469 | 0.621 | 0.576 | 0.763 | 0.683 |
| S1 | - | 0.473 | 0.698 | 0.503 | 0.614 | 0.545 | 0.795 | 0.682 |
| S2 | - | 0.441 | 0.659 | 0.508 | 0.408 | 0.555 | 0.772 | 0.686 |
| UT (T1–T4) | 0.667 | 0.657 | 0.779 | 0.673 | 0.749 | 0.741 | 0.840 | 0.788 |
| MT (T5–T8) | 0.577 | 0.685 | 0.772 | 0.646 | 0.688 | 0.702 | 0.823 | 0.770 |
| LT (T9–T12) | 0.601 | 0.677 | 0.817 | 0.609 | 0.699 | 0.739 | 0.804 | 0.784 |
| TL (T11–L2) | 0.633 | 0.706 | 0.828 | 0.645 | 0.630 | 0.701 | 0.808 | 0.787 |
| UL (L1–L2) | 0.592 | 0.701 | 0.789 | 0.635 | 0.585 | 0.676 | 0.803 | 0.768 |
| LL (L3–L5) | 0.433 | 0.596 | 0.799 | 0.533 | 0.618 | 0.652 | 0.795 | 0.738 |
| SA (S1–S2) | - | 0.458 | 0.647 | 0.545 | 0.547 | 0.567 | 0.798 | 0.694 |
| EC (T5+T6+L1) | 0.602 | 0.711 | 0.805 | 0.684 | 0.738 | 0.731 | 0.836 | 0.792 |
| TS (T1-S2) | 0.608 | 0.686 | 0.816 | 0.700 | 0.754 | 0.749 | 0.842 | 0.799 |

All reported correlation coefficients were statistically significant (p < 0.002).
