# Supplementary material for: Which vertebral levels provide practical HU-based assessment for osteoporosis screening? A comparative analysis from T1 to S2
Source: J Orthop Surg Res. 2026 Jun 23;21:565. doi: 10.1186/s13018-026-06952-8 (PMC13621712; doi:10.1186/s13018-026-06952-8)
Supplement: Supplementary file 3 [file 13018_2026_6952_MOESM3_ESM.docx]

Supplementary Table 3. Diagnostic performance indicators of single-segment HU for osteoporosis prediction

| Segment | AUC (95% CI) | cut-off (HU) | Sensitivity (%) | Specificity (%) | p-value |
| --- | --- | --- | --- | --- | --- |
| T1 | 0.887 (0.850-0.923) | 182.5 | 95.00 | 66.82 | <0.001 |
| T2 | 0.878 (0.839-0.916) | 167.5 | 96.25 | 63.18 | <0.001 |
| T3 | 0.888 (0.851-0.924) | 147.5 | 87.50 | 73.64 | <0.001 |
| T4 | 0.887 (0.851-0.924) | 139.5 | 92.50 | 72.27 | <0.001 |
| T5 | 0.901 (0.868-0.935) | 135.5 | 96.25 | 69.55 | <0.001 |
| T6 | 0.888 (0.852-0.924) | 131.5 | 98.75 | 66.82 | <0.001 |
| T7 | 0.874 (0.835-0.912) | 108.5 | 87.50 | 75.91 | <0.001 |
| T8 | 0.875 (0.836-0.913) | 105.5 | 86.25 | 74.55 | <0.001 |
| T9 | 0.878 (0.840-0.916) | 117.5 | 92.50 | 69.55 | <0.001 |
| T10 | 0.896 (0.861-0.931) | 105.2 | 78.75 | 85.00 | <0.001 |
| T11 | 0.912 (0.880-0.943) | 100.5 | 88.75 | 83.64 | <0.001 |
| T12 | 0.911 (0.880-0.943) | 99.5 | 96.25 | 77.27 | <0.001 |
| L1 | 0.900 (0.866-0.934) | 83.5 | 90.00 | 79.55 | <0.001 |
| L2 | 0.891 (0.856-0.927) | 85.5 | 95.00 | 72.73 | <0.001 |
| L3 | 0.886 (0.849-0.923) | 64.5 | 87.50 | 80.45 | <0.001 |
| L4 | 0.899 (0.865-0.934) | 73.5 | 91.25 | 80.00 | <0.001 |
| L5 | 0.897 (0.861-0.933) | 79.5 | 90.00 | 83.18 | <0.001 |
| S1 | 0.887 (0.847-0.926) | 118.5 | 92.50 | 77.73 | <0.001 |
| S2 | 0.862 (0.821-0.904) | 75.5 | 86.25 | 76.36 | <0.001 |
